# Nutritional counseling tailored to the patient’s learning type and its impact on interdialytic weight gain in chronic hemodialysis patients

**DOI:** 10.1590/2175-8239-JBN-2023-0205en

**Published:** 2025-03-10

**Authors:** Naiane Rodrigues de Almeida, Caio Pellizzari, Cynthia Leinig, Fabiana Nerbass, Thyago Proença de Moraes

**Affiliations:** 1 Pontifícia Universidade Católica do Paraná, Curitiba, PR, Brazil.; 2 Santa Casa de Misericórdia de Curitiba, Curitiba, PR, Brazil.; 3 Fundação Pró Rim, Joinville, SC, Brazil.

**Keywords:** VARK, Dialysis, Hypervolemia

## Abstract

**Introduction:**

Interdialytic weight gain (IDWG) is a risk factor for cardiovascular complications and mortality in hemodialysis (HD) patients. Sodium and fluid intake are important modifiable risk factors for these outcomes, and the role of the nutritionist as educator is essential in this process. However, patient adherence is often low, representing a significant challenge. Recent recommendations suggest personalizing the education process, but there is limited data on how to effectively carry out this adaptation. The present study aimed to assess the effectiveness of a personalized nutritional intervention based on learning style using the VARK tool with the goal of reducing IDWG in HD patients.

**Objective:**

This was a randomized crossover clinical trial. Patients in the intervention group received individualized nutritional guidance based on their learning style, as determined by the VARK questionnaire. The control group received standard guidance without any personalization or educational materials.

**Results:**

A total of 21 chronic HD patients participated in the study. On average, the baseline IDWG in the population was 2.61 ± 1.08 liters, and we did not observe any significant pattern of change in either IDWG (p = 0.55) or systolic blood pressure (p = 0.44) over the study period.

**Conclusion:**

Tailored nutritional counseling based on the patient’s learning style did not lead to an improvement in IDWG in our population.

## Introduction

Interdialytic weight gain (IDWG) is routinely used as a parameter for fluid intake between hemodialysis sessions. The higher the IDWG, the higher the pre-dialysis blood pressure (BP), the risk of hemodynamic instability during the hemodialysis session, and naturally, the risk of mortality^1^.

Different factors can contribute to IDWG, including age, gender, primary renal disease, residual renal function, and salt/fluid intake. The latter is the main modifiable factor, and dietary counseling plays an important role in raising patient awareness about the importance of fluid and sodium restriction. However, given the multiple dietary restrictions for patients on dialysis, these are difficult to achieve despite the hard work of dietitians, nurses and doctors. Salt intake is a particular problem. In Brazil, for cultural and social reasons, the salt intake in the general population is twice the amount recommended by the World Health Organization (5 g/day)^2^. According to a study by Nerbass et al.^3^, dialysis patients had a salt intake of 8.6 g/day in prevalent hemodialysis patients.

The way we educate patients may have impact on patient participation and adherence to treatment. In our setting, dialysis patients are regularly educated using a standard approach, despite having different needs. Individualization of treatment is recommended by guidelines, but details on patient education are lacking. The VARK system characterizes the preferred way of learning for people. Whether an approach to reduce IDWG focuses on the patient’s learning style is effective is unknown. Therefore, we designed a cross-over study to analyze IDWG before and after counselling tailored to learning style.

## Methods

**Figure 1:**
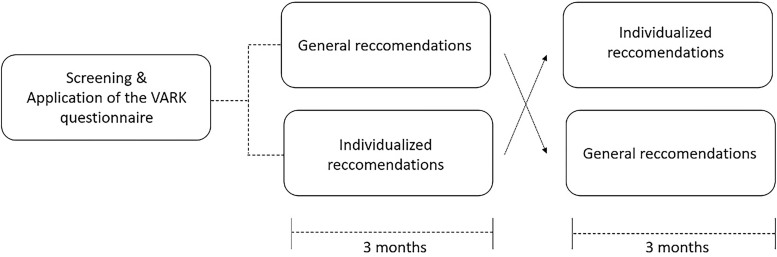
Study design.

This was a single center, randomized crossover trial to evaluate the impact of a dietitian orientation tailored to the patient’s learning type to reduce interdialytic weight gain in chronic hemodialysis patients (Figure 1). The study was approved by the local ethics committee and all patients signed the consent form before inclusion in the study (n° 4.601.788). Patients were selected from a single dialysis center from the university hospital Santa Casa de Misericórdia de Curitiba, Paraná, Brazil.

The clinic attends 120 chronic HD patients and 30 chronic peritoneal dialysis patients. After identifying the potentially eligible candidates, we contacted all patients to explain the protocol, invite them to join the study, and obtain their signature for informed consent.

The inclusion criteria were adults > 18 years old on chronic hemodialysis for at least 3 months and willing to sign the informed consent. The exclusion criteria were individuals with pacemaker, metallic cardiac valves, limb amputations, visual impairment, and illiteracy. The sample size was calculated aiming a 25% reduction in the interdialytic weight gain with an alpha level of 0.05 and a power of 80%.

The formula used was $n={\left(\frac{Z\alpha }{2}+Z\beta \right)}^{2}*2*\sigma^{2}/\Delta^{2}$

where *n* is the sample size, Z = 1.96 when alpha equals 0.05, Zβ = 0.84 (the normal value for a power of 80%), σ is the standard deviation, and delta is the mean difference between the groups.

### Bioimpedance Spectroscopy

To evaluate volemic status, we used a multifre­quency bioimpedance device (Body Composition Monitor – BCM^®^ – Fresenius^®^ Medical Care, Deutschland GmbH, Germany).

### Type of Learning

**Figure 2:**
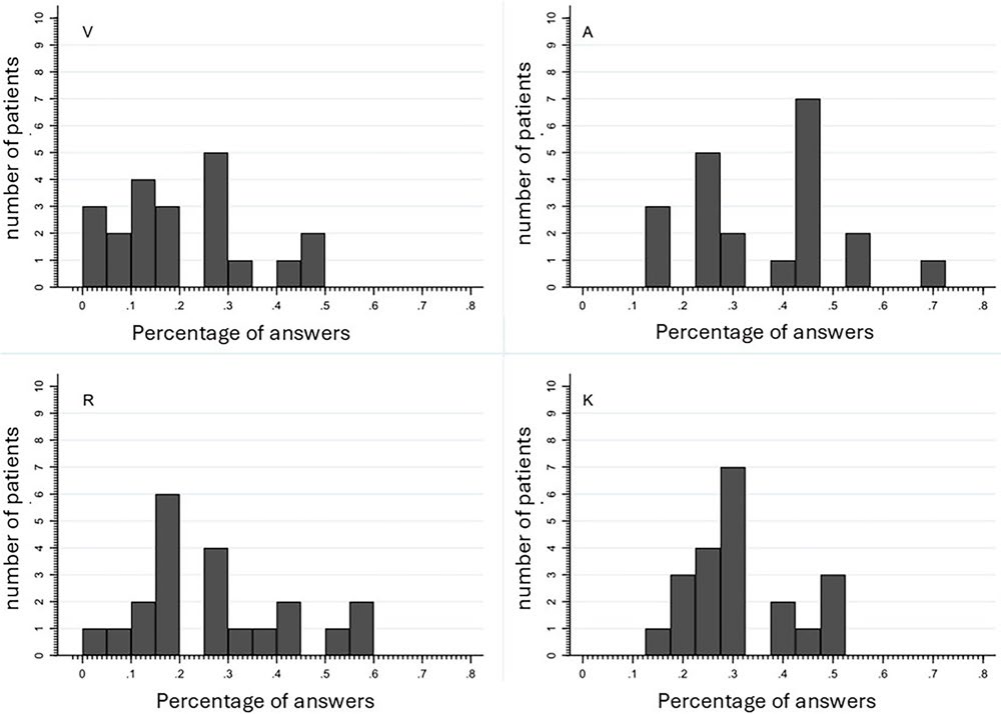
VARK score distribution for each learning type.

We identified the preferred way of learning of all patients included in the study using a tool known as VARK (Visual – Auditive – Reading – Kinesthetic). The VARK questionary comprises 16 questions with 4 possible answers. Each answer is associated with one type of learning preference and respondents can choose one or more answers for each question (see supplementary file for questions and answers of the VARK questionnaire). We grouped the study participants into V, A, R, or K categories based on the type of learning that had the highest score. Additionally, we also classified patients as having a bimodal preference if they selected 2 of the 4 learning types more than 25% of the time and as having a multimodal preference if they selected 3 or 4 learning types (as shown in Figure 2).

### Blood Pressure Measurements

Blood pressure was measured immediately before the dialysis session, following all best practice recom­mendations regarding patient positioning and cuff size, over the 60-day study period, with 30 days in the first phase and 30 in the second phase. We did not measure blood pressure during the washout period. Importantly, the study took place during the COVID-19 pandemic but no adjustments or changes in therapy duration and frequency occurred during this period.

### Intervention

We developed four tools for individualized patient education based on the primary type of learning of each individual (as shown in Table 1). Each training session lasted 20 to 30 minutes for every patient and was conducted in 2 sessions (during dialysis) addressing topics such as: concepts related to fluid overload, importance of adhering to nutritional guidelines, and dietary guidance on sodium and fluid intake. In the standard follow-up, a brief verbal nutritional guidance was provided without the use of educational materials as a reinforcement regarding fluid and sodium restriction (Figure 1).

**Table 1:** Educational material

	Material developed
Visual	• Infographic The topic was presented in an infographic format that emphasizes visual elements such as images, shapes, flowcharts, color highlights, and organized visuals with alternate fonts and symbols. The content was presented on a tablet and also distributed in a brochure format with full-color printing. • “FLASH CARDS”. An image is shown on the front of the card and the patient is asked to deduce the information on the back. For example, a food is show on the front, while on the back, a red or green sign indicates the sodium content (red = high and green = low). For liquids, the front shows a photo of a liquid in container and the back provides information about the equivalent amount in 200-ml glasses.
Aural/auditory	• Explanation of concepts and guidance in an oral manner. During the presentation, questions about the topic are asked to the patient to encourage the patient to speak and interact. • At the end, an oral test with questions and answers about the guidance that was provided is conducted, so that the patient can repeat the concepts out loud. • An audio message about the topic and guidance (available on messaging apps) is sent for later reference.
Read/Write	• Written guidance is provided in A4 paper. Initially presented on a tablet, and at certain moments, the patient is asked to read some highlights out loud. • The written material is provided in A4 paper for the patient to read. • In the written material, questions about the topic for the patient to write down the answers are included, promoting both reading and writing skills.
Kinesthetic	• Presentation of concepts using PowerPoint, with the use of photos and videos of real-life situations to exemplify foods/volume of liquids, edema, etc. • Food labels are shown for evaluation and comparison of sodium content in the nutrition table. Examples of foods that are low and high in sodium are provided. • Containers of different sizes are used to develop the concept of quantity (cup, mug, bowl). The patient is asked to try to guess how many mL each container contains. • Upon completion, with the participation of the patient, the number of glasses of water needed (hypothetically) to achieve the weight gain presented since the last dialysis session is calculated.

### Statistics

Continuous variables were reported as mean ± standard deviation or median and interquartile range depending on data distribution, and categorical variable as percentage and absolute number. The comparison of clinical parameters between groups (based on type of intervention) was performed using a multilevel mixed-effects linear regression, where all clinical measurements were at the first level and the patient at the second level. We used STATA 17^®^ for all analysis.

## Results

Our study included 21 chronic hemodialysis patients with mean age of 54.3 ± 14.4 years, 24% male, 24% with diabetes, and 81% with diagnosis of hypertension. Details on all demographic and clinical characteristics can be found in Table 2.

**Table 2:** Clinical and demographical characteristics of the study population

Variable	Result
Demographic	
Age (years)	54.3 ± 14.4
Diabetes (yes)	23.8%
Hypertension (yes)	80.9%
Incomes	
1	19.0%
2	42.9%
3	28.6%
4	9.5%
Literacy	
1	28.6%
2	47.6%
3	23.8%
Male gender	23.8%
Previous hemodialysis (yes)	19.0%
VARK preference	
2	47.6%
3	14.3%
4	23.8%
5	14.3%
Clinic	
Dialysis vintage (months)	20 (11–41)
SBP (mmHg)	166.6 ± 17.3
Ultrafiltration (liters)	2.7 ± 1.0
Laboratory	
Potassium (mEq/L)	4.9 ± 0.8
Albumin (g/dl)	3.9 ± 0.4

The majority of patients had a combination of responses across the four learning types, with very few having over 50% of their answers within a single learning type (Figure 2). Only 7 patients obtained more than 50% of answers within at least one type of learning and 3 of them were classified as bimodal patients (1 visual/reading, 1 auditory/reading, and 1 auditory/kinesthetic). If we considered only the variable with the highest value to categorize our subjects (regardless of the 50% threshold) then we have: 10 patients as auditory, 3 as reading, 5 as kinesthetic, and 3 with a bimodal type of learning.

**Figure 3:**
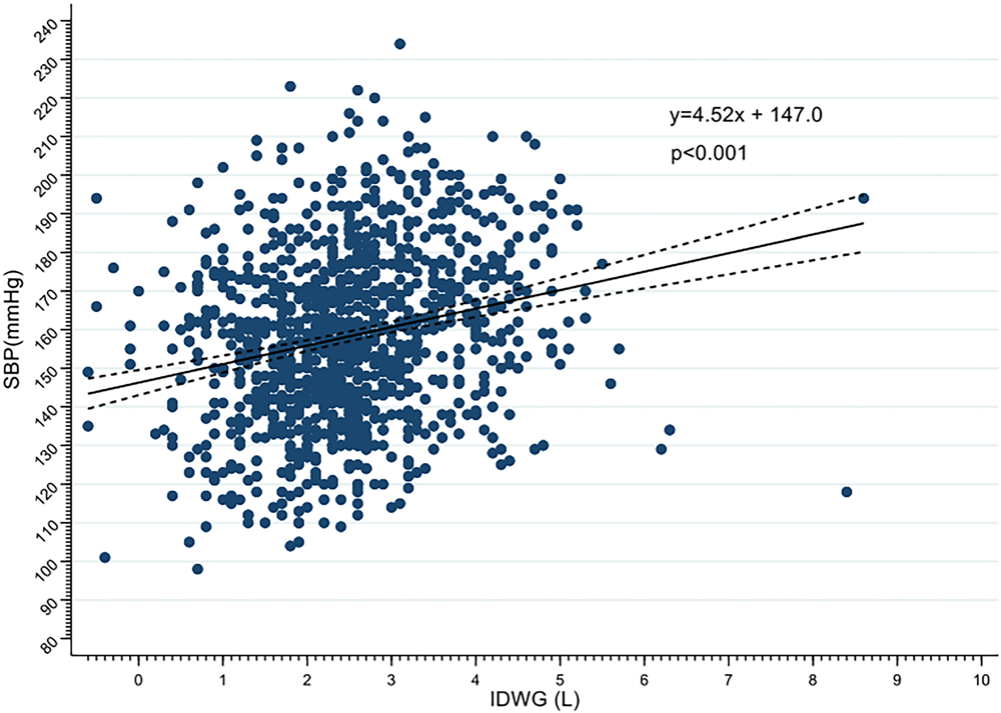
Correlation between blood pressure and IDWG.

**Figure 4:**
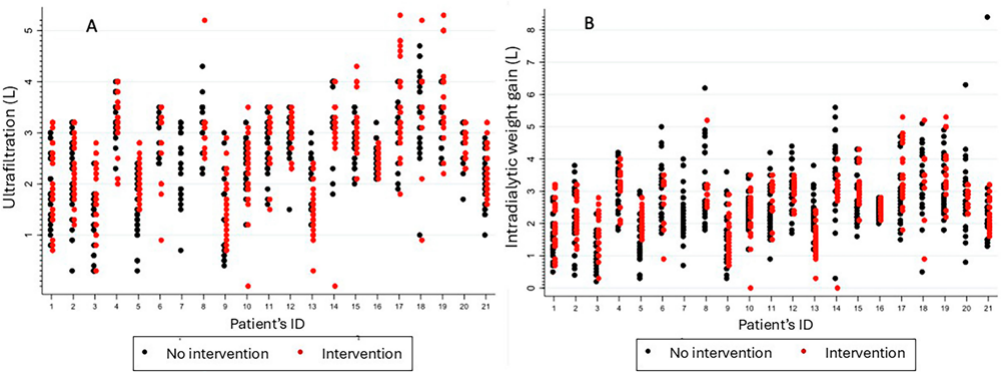
A. Ultrafiltration before and after intervention per patient; B. Intradialytic weight gain before and after intervention per patient.

The blood pressure of study population was high compared to the current standards. Of the 1,155 measurements performed in all 21 patients, 91.9% were above 120 mmHg, 85.4% were above 130 mmHg, and 74.5% were above 140 mmHg. Systolic blood pressure (SBP) positively correlated with IDWG (Figure 3) with an increase of 4.5 mmHg for each 1-liter increase in IDWG. On average, the baseline IDWG (first 3 measures) of the population was 2.61 ± 1.08 liters (Figure S1) and no significant change was found both in IDWG and SBP over the study period (Figure 4A and 4B). In addition, no change related to the intervention was found on DBP, session Kt/V, and overhydration (in liters) measured with multifrequency bioimpedance (Table 3). There were no changes in the prescription of antihypertensive medication between the groups along the study period.

Notes – Figure A depicts individual values of ultrafiltration during the study before and after intervention. The figure B depicts individual values of IDWG during the study before and after intervention.

**Table 3:** Clinical parameters of the groups with and without individualized education

Parameters	Standard	Individualized	p
Post dialysis systolic blood pressure (mmHg)	157.1 ± 17.2	159.5 ± 16.2	0.56
Pre-dialysis systolic blood pressure (mmHg)	153.7 ± 16.3	152.0 ± 16.1	0.44
KtV	1.55 ± 0.56	1.61 ± 0.27	0.52
Interdialytic weight gain (kg)	2.48 ± 0.69	2.53 ± 0.62	0.55
Ultrafiltration per session (liters)	2.59 ± 0.71	2.70 ± 0.69	0.24
***Bioimpedance spectroscopy***			
Overhydration (liters)	1.5 ± 2.0	1.41 ± 1.68	0.97

**Figure 5:**
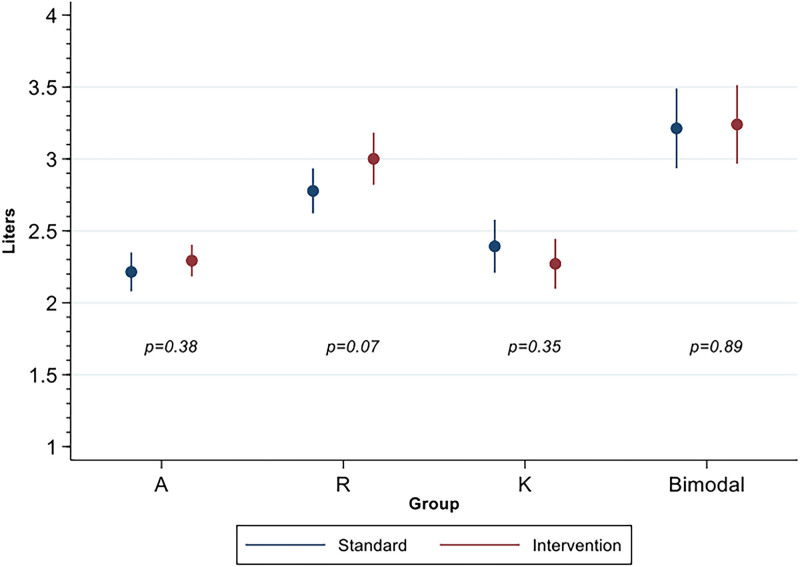
Interdialytic weight gain before and after intervention stratified by learning type.

We also stratified our population to explore our results according to the specific type of learn­ing (Figure 5). The intervention was found to be ineffective regardless of the type of learning.

Abbreviations – A: auditive; R: reading; K: kinaesthetic. No patient was classified as a visual learner.

## Discussion

This study is the first to our knowledge to test the efficiency of an individualized nutritional orientation developed for different learning types in reducing IDWG in chronic hemodialysis patients. Our results suggest that this tool was ineffective to reduce IDWG in the short term.

IDWG is an easily identifiable clinical sign in clinical practice and correlates well with high blood pressure and hypervolemia, which in turn, are known predictors of cardiovascular events and poor quality of life^4^. A study involving a large cohort of over 38,000 patients found that the mortality rate among patients within the highest quartile of IDWG was more than 2.5 times higher than that of the 2nd quartile^5^. The group with the highest mortality rate had an IDWG of over 4 kg, compared to 2.3 kg in the 2nd quartile group. The mean IDWG of our population was somewhere between the 2nd and 3rd quartile of the study of Hecking et al.^5^ which had a 17 to 20% higher risk of death. This high-risk characteristic of our population prompted us to seek for a strategy to lower IDWG.

IDWG is primarily influenced by the patient’s compliance with the medical and multidisciplinary team’s dietary recommendations regarding sodium and fluid intake. Given this premise, multiple authors emphasize the importance of tailoring the approach to patients on dialysis in order to minimize weight gained between sessions^6,7^. The idea is that this individualized approach may increase patient adherence to therapy. However, there is no consensus on how best to personalize this recommendation.

We selected the VARK questionnaire as a tool for imparting knowledge in a personalized manner. The instrument was first applied to a group of students and has an estimated reliability of 80%^8^. The VARK system categorizes individuals based on their preferred method of receiving new information – visual, auditory, reading/writing, kinesthetic, or multimodal – and was found to be useful when applied in medical sciences^9^. In hypertensive patients with uncontrolled BP, a better long-term BP control was achieved after individualized intervention using the VARK system with a mean reduction of 9 mmHg in systolic BP^10^. In another study with diabetics, the group that used the individualized strategy achieved greater success in disease-related self-care compared to the conventional strategy^11^. However, like the current study, not all research has shown positive results – Chan et al. found no improvement in treatment adherence using a visual device for asthma management in individuals with a visual learning preference^12^.

The reasons for the differing outcomes in these studies with diverse populations are not immediately apparent and require a more in-depth examination. For instance, our patients had a chronic condition that leads to various limitations, and therefore, breaking habitual routines is difficult with a one-time intervention. In addition, other factors affect treatment adherence, such as gender, educational level, short dialysis time^10^, age, low health literacy, and low quality of life^13^. A limited health literacy of 22.7% (CI: 95% 20.6–24.8%) was found in a systematic review with 1405 individuals, which was independently associated with socioeconomic factors such as lower education level, income, and worse health outcomes^14^. As health literacy has important implications, interventions for the educational process require an individualized and continuous approach, as well as consideration of the specific learning style of the patient. It is likely that the unsuccessful results found here were due in part to the unique approach of the study. Nonetheless, it is recognized in the medical community that our conventional (one size fits all) approach needs to be re-evaluated^15^.

A meta-analysis with 40 randomized clinical trials and a total of 2278 patients showed that psychosocial and educational interventions raised the adherence rates of patients in hemodialysis and peritoneal dialysis, and were evident in the IDWG and dry weight/IDWG ratio. The interventions were based on different contexts, such as cognitive-behavioral therapy, motivational interviewing, relaxation, support, education, counseling, consul­tation, provision of resources, supervision, and direct telephone contact^16^. The study showed that treatment adherence is related to multiple factors that contribute to patients adopting behaviors, which was not possible to observe in our study.

The approach to improving treatment adherence in chronic kidney disease patients must include knowledge, behaviors, actions, and skills to empower them to take responsibility for their own care. In this way, the information provided must be meaningful, take into account their learning style, and be broken down into steps that can be achieved in small, short-term goals. This will increase confidence so that the patient feels capable to master that new knowledge and put it into practice^17^.

Our study had some important limitations that must be taken into account, including the fact that it was a single center study with a small number of patients, that the frequency of the intervention might not be sufficient to increase awareness, that all patients were already on dialysis, which makes it harder to change their behavior as they may have been following a certain routine, and that only one trained person (a dietitian) carried out the intervention, which can be both positive, as there was no interobserver variation, and negative, as there may have been problems with the person performing the intervention. Furthermore, although illiterate patients were excluded, it is not known whether the degree of health literacy was sufficient to fully understand the advice.

In conclusion, IDWG remains a serious problem in the hemodialysis population. There seems to be an agreement on individualization of treatment and nutritional counseling, but how to best individualize treatment is still unknown. Treatment individualization based on the patient’s learning style did not improve IDWG in our population.

## Supplementary Material

The following online material is available for this article:

VARK Questionnaire.

Figure S1 – IDWG at baseline.
