# Supplementary material for: Nutritional counseling tailored to the patient’s learning type and its impact on interdialytic weight gain in chronic hemodialysis patients
Source: J Bras Nefrol. 2025 Mar 10;47(2):e20230205. doi: 10.1590/2175-8239-JBN-2023-0205en (PMC11913451; doi:10.1590/2175-8239-JBN-2023-0205en)
Supplement: Supplementary file 1 [file 2175-8239-jbn-47-2-e20230205-suppl1.pdf]

Braz. J. Nephrol.
https://doi.org/10.1590/2175-8239-JBN-2023-0205en

Supplementary Material to "Nutritional counseling tailored for patient's learning type and its impact on interdialytic weight gain in chronic hemodialysis patients"

VARK Questionnaire

I would like to know about a new project. I ask for:

an opportunity to discuss the project.

diagrams showing the phases of the project with tables of benefits and costs.

a written report describing the key features of the project.

examples where the project has been successfully implemented.

When learning from the internet I like:

interesting design and visual features.

videos that show how to do or make something.

audio channels where I can listen to podcasts or interviews.

interesting written descriptions, lists and explanations.

I would like to learn more about a tour I will participate in. I would:

read the information about the tour in the itinerary.

look at details of the tour highlights and activities.

use a map and see where the places are.

talk to the person who planned the tour or others who are taking part in the tour.

I have a problem with my heart. I would prefer the doctor to

described what is going on.

gave me something to read to explain what is wrong.

showed me a diagram of what was wrong.

showed me what was wrong using a plastic model.

I prefer a lecturer or teacher who:

diagrams, tables, maps or charts.

handouts, books or readings.

questions and answers, lectures, group discussions or guest speakers.

demonstrations, models or practical exercises.

I have completed a competition or test and would like feedback. I would like feedback:

using examples that I have done.

by means of diagrams showing what I have achieved.

by means of a written description of my results.

from someone who talks it through with me.

I would like to learn how to take better photos. I would:

use examples of good and bad photos and show how to improve them.

ask questions and talk about the camera and its functions.

use written instructions on what to do.

use diagrams showing the camera and the functions of each part.

When I learn, I use:

use examples and applications.

i see patterns in things.

i like to talk things through.

i read books, articles and handouts.

I would like to assemble a wooden table that came in pieces (kit). I would learn best by:

written instructions that came with the parts for the table.

advice from someone who has done it before.

watching a video of someone assembling a similar table.

diagrams showing each step of the assembly.

I would like to learn how to play a new board or card game. I would:

read the instructions.

use the diagrams that explain the different phases, moves and strategies of the game.

watch others play before I join in.

listen to someone explain it to me and ask questions.

I would like to learn to do something new on the computer. I would:

read the written instructions that come with the program.

follow the diagrams in a book.

start using the program and learn by trial and error.

talk to people who are familiar with the program.

I would like to save more money and decide between different options. I would:

review examples of each option based on my financial data.

read a printed brochure detailing the options.

talk to an expert about the options.

use charts showing different options for different time periods.

I want to find out about a house or apartment. Before I view it, I would like to:

a plan showing the rooms and a map of the area.

a printed description of the rooms and facilities.

a conversation with the owner.

watch a video of the property.

When choosing a career or field of study, these points are important to me:

Applying my knowledge in real-life situations.

Working with designs, maps or diagrams.

Communicating with others through discussion.

Being good with words in written communication.

Finding my way to a business that a friend recommended. I would:

ask my friend to tell me the way.

find out where the store is in relation to a place I know.

use a map.

write down the directions I need to remember.

There is a video on a website that shows how to make a special chart or table. There is a person talking, some lists and words describing what to do, and some diagrams. I would learn the most by:

watching the actions.
