# Supplementary material for: Nutritional counseling tailored to the patient’s learning type and its impact on interdialytic weight gain in chronic hemodialysis patients
Source: J Bras Nefrol. 2025 Mar 10;47(2):e20230205. doi: 10.1590/2175-8239-JBN-2023-0205en (PMC11913451; doi:10.1590/2175-8239-JBN-2023-0205en)
Supplement: Supplementary file 2 [file 2175-8239-jbn-47-2-e20230205-suppl2.pdf]

Braz. J. Nephrol.
https://doi.org/10.1590/2175-8239-JBN-2023-0205en

Supplementary Material to “Nutritionist’s counseling tailored for patient’s learning type and its impact on interdialytic weight gain of chronic hemodialysis patients”

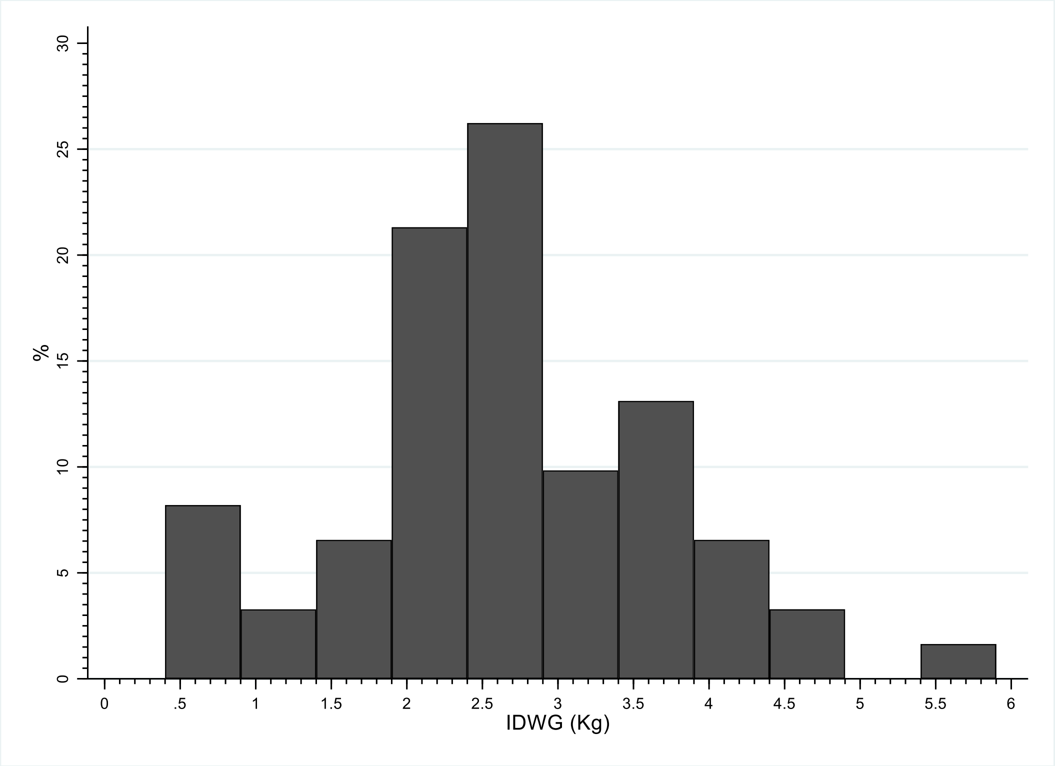

Figure S1 - IDWG at baseline.
