# Supplementary material for: Nutritional counseling tailored to the patient’s learning type and its impact on interdialytic weight gain in chronic hemodialysis patients
Source: J Bras Nefrol. 2025 Mar 10;47(2):e20230205. doi: 10.1590/2175-8239-JBN-2023-0205en (PMC11913451; doi:10.1590/2175-8239-JBN-2023-0205en)
Supplement: Supplementary file 3 [file 2175-8239-jbn-47-2-e20230205-suppl3.pdf]

Braz. J. Nephrol.
https://doi.org/10.1590/2175-8239-JBN-2023-0205pt

Material Suplementar para "Orientação nutricional personalizada segundo o tipo de aprendizagem do paciente e seu impacto no ganho de peso interdialítico em pacientes crônicos em hemodiálise"

Questionário VARK

Gostaria de saber sobre um novo projeto. Solicito:
uma oportunidade de discutir o projeto.
diagramas mostrando as fases do projeto com tabelas de benefícios e custos.
um relatório por escrito descrevendo as principais características do projeto.
exemplos de implementação bem-sucedida do projeto.

Quando aprendo pela Internet, gosto de:
design e recursos visuais interessantes.
vídeos que mostram como fazer ou criar algo.
canais de áudio onde posso ouvir podcasts ou entrevistas.
descrições, listas e explicações interessantes por escrito.

Gostaria de saber mais sobre um passeio do qual participarei. Gostaria de:
ler as informações sobre o passeio no itinerário.
ver detalhes dos destaques e atividades do passeio.
usar um mapa e ver onde ficam os lugares.
conversar com a pessoa que planejou o passeio ou com outras pessoas que estejam participando do passeio.

Tenho um problema no coração. Eu preferiria que o médico:
descrevesse o que está acontecendo.
me desse algo para ler que explicasse o problema.
mostrasse um diagrama sobre o que há de errado.
mostrasse o que há de errado utilizando um modelo de plástico.

Prefiro um palestrante ou professor que utilize:

diagramas, tabelas, mapas ou gráficos.

apostilas, livros ou leituras.

perguntas e respostas, palestras, discussões em grupo ou palestrantes convidados.

demonstrações, modelos ou exercícios práticos.

Concluí uma competição ou teste e gostaria de receber feedback. Gostaria de recebê-lo:

por meio de exemplos do que fiz.

por meio de diagramas que mostrem o que consegui.

por meio de uma descrição escrita de meus resultados.

de alguém que converse comigo sobre o assunto.

Gostaria de aprender a tirar fotos melhores. Eu gostaria de:

usar exemplos de fotos boas e ruins e mostrar como melhorá-las.

fazer perguntas e falar sobre a câmera e suas funções.

usar instruções escritas sobre o que fazer.

usar diagramas que mostrem a câmera e as funções de cada parte.

Quando aprendo, utilizo:

exemplos de uso e aplicativos.

vejo padrões nas coisas.

gosto de conversar sobre as coisas.

leio livros, artigos e apostilas.

Gostaria de montar uma mesa de madeira que veio em peças (kit). Eu aprenderia melhor com:

instruções escritas que acompanham as peças da mesa.

orientação de alguém que já fez isso antes.

um vídeo de alguém montando uma mesa semelhante.

diagramas mostrando cada etapa da montagem.

Gostaria de aprender a jogar um novo jogo de tabuleiro ou de cartas. Gostaria de:

ler as instruções.

usar diagramas que explicam as diferentes fases, movimentos e estratégias do jogo.

observar os outros jogando antes de participar.

ouvir alguém me explicar e tirar dúvidas.

Gostaria de aprender a fazer algo novo no computador. Eu gostaria de:
ler as instruções escritas que acompanham o programa.
seguir os diagramas de um livro.
começar a usar o programa e aprender por tentativa e erro.
conversar com pessoas que estejam familiarizadas com o programa.

Gostaria de economizar mais dinheiro e decidir entre diferentes opções. Eu gostaria de:
analisar exemplos de cada opção com base em meus dados financeiros.
ler um folheto impresso detalhando as opções.
conversar com um especialista sobre as opções.
utilizar gráficos que mostrem diferentes opções para diferentes períodos de tempo.

Quero saber mais sobre uma casa ou apartamento. Antes de vê-lo, gostaria de:
uma planta mostrando os cômodos e um mapa da área.
uma descrição impressa dos quartos e das instalações.
uma conversa com o proprietário.
assistir a um vídeo da propriedade.

Ao escolher uma carreira ou área de estudo, estes pontos são importantes para mim:
aplicar meus conhecimentos em situações de vida real.
trabalhar com projetos, mapas ou diagramas.
comunicação com outras pessoas por meio de conversas.
ser bom com palavras na comunicação escrita.

Encontrar o caminho para um negócio recomendado por um amigo. Eu gostaria de:
pedir a meu amigo que me indique o caminho.
descobrir onde fica a loja em relação a um lugar que conheço.
utilizar um mapa.
escrever as instruções que preciso lembrar.

Há um vídeo em um site que mostra como criar um gráfico ou uma tabela especial. Há uma pessoa falando, algumas listas e palavras descrevendo o que fazer e alguns diagramas. Eu aprenderia mais:
ouvindo
observando as ações.
lendo as palavras
olhando os diagramas
