# Supplementary material for: Nutritional counseling tailored to the patient’s learning type and its impact on interdialytic weight gain in chronic hemodialysis patients
Source: J Bras Nefrol. 2025 Mar 10;47(2):e20230205. doi: 10.1590/2175-8239-JBN-2023-0205en (PMC11913451; doi:10.1590/2175-8239-JBN-2023-0205en)
Supplement: Translation

# Translation: Orientação nutricional personalizada segundo o tipo de aprendizagem do paciente e seu impacto no ganho de peso interdialítico em pacientes crônicos em hemodiálise

**DOI:** 10.1590/2175-8239-JBN-2023-0205pt

**Authors:** Naiane Rodrigues de Almeida, Caio Pellizzari, Cynthia Leinig, Fabiana Nerbass, Thyago Proença de Moraes

**Affiliations:** 1 Pontifícia Universidade Católica do Paraná, Curitiba, PR, Brasil.; 2 Santa Casa de Misericórdia de Curitiba, Curitiba, PR, Brasil.; 3 Fundação Pró Rim, Joinville, SC, Brasil.

**Keywords:** VARK, Diálise, Hipervolemia

## Abstract

**Introdução:**

O ganho de peso interdialítico (GPID) é um fator de risco para complicações cardiovasculares e mortalidade em pacientes em hemodiálise (HD). A ingestão de sódio e líquidos é um importante fator de risco modificável para esses desfechos, e o papel do nutricionista como educador é essencial nesse processo. Entretanto, a adesão dos pacientes costuma ser baixa, o que representa um desafio significativo. Recomendações recentes sugerem a personalização do pro­cesso educacional, mas existem dados limitados sobre como realizar essa adaptação de forma eficaz. O presente estudo teve como objetivo avaliar a eficácia de uma intervenção nutricional personalizada baseada no estilo de aprendizagem, utilizando a ferramenta VARK, a fim de reduzir o GPID em pacientes em HD.

**Métodos:**

Este foi um ensaio clínico randomizado cruzado. Os pacientes do grupo intervenção receberam orientação nutricional individualizada com base em seu estilo de aprendizagem, conforme determinado pelo questionário VARK. O grupo controle recebeu orientação padrão, sem qualquer personalização ou materiais educacionais.

**Resultados:**

Participaram do estudo 21 pacientes crô­nicos em HD. Em média, o GPID basal na população foi de 2,61 ± 1,08 litros, e não observamos nenhum padrão signi­ficativo de alteração no GPID (p = 0,55) ou na pressão arterial sistólica (p = 0,44) durante o período do estudo.

**ConclusÃO:**

A orientação nutricional personalizada com base no estilo de aprendizagem do paciente não resultou em uma melhora no GPID em nossa população.

## Introdução

O ganho de peso interdialítico (GPID) é utilizado rotineiramente como parâmetro para a ingestão de líquidos entre as sessões de hemodiálise. Quanto maior o GPID, maior a pressão arterial (PA) pré-diálise, o risco de instabilidade hemodinâmica durante a sessão de hemodiálise e, naturalmente, o risco de mortalidade^1^.

Diferentes fatores podem contribuir para o GPID, incluindo idade, sexo, doença renal primária, função renal residual e ingestão de sal/fluidos. Este último é o principal fator modificável, e a orientação dietética desempenha um papel importante no aumento da conscientização dos pacientes sobre a importância da restrição de fluidos e sódio. No entanto, devido às múltiplas restrições alimentares para pacientes em diálise, torna-se difícil alcançá-las, apesar do trabalho árduo de nutricionistas, enfermeiros e médicos. O consumo de sal é um problema específico. No Brasil, por razões culturais e sociais, a ingestão de sal na população geral é o dobro do recomendado pela Organização Mundial da Saúde (5 g/dia)^2^. De acordo com um estudo realizado por Nerbass et al.^3^, pacientes prevalentes em hemodiálise apresentaram uma ingestão de sal de 8,6 g/dia.

A forma como educamos os pacientes pode ter impacto em sua participação e adesão ao tratamento. Em nosso contexto, pacientes em diálise são regularmente instruídos utilizando uma abordagem padrão, apesar de apresentarem necessidades diferentes. A individualização do tratamento é recomendada pelas diretrizes, mas não há detalhes sobre a educação do indivíduo. O sistema VARK caracteriza a forma preferida de aprendizado das pessoas. Não se sabe se uma abordagem para reduzir o GPID com foco no estilo de aprendizagem do paciente é eficaz. Portanto, desenhamos um estudo cruzado para analisar o GPID antes e após a orientação personalizada de acordo com o estilo de aprendizagem.

## Métodos

Este foi um estudo cruzado, randomizado e de centro único para avaliar o impacto da orientação nutricional feita por um nutricionista, personalizada de acordo com o tipo de aprendizagem do paciente, para reduzir o ganho de peso interdialítico em pacientes crônicos em hemodiálise (Figura 1). O estudo foi aprovado pelo Comitê de Ética local e todos os pacientes assinaram o termo de consentimento antes da inclusão no estudo (n° 4.601.788). Os pacientes foram selecionados a partir de um único centro de diálise do hospital universitário Santa Casa de Misericórdia de Curitiba, Paraná, Brasil.

**Figura 1:** Desenho do estudo.

A clínica atende 120 pacientes crônicos em HD e 30 pacientes crônicos em diálise peritoneal. Após identificar os candidatos potencialmente elegíveis, entramos em contato com todos os pacientes para explicar o protocolo, convidá-los a participar do estudo e obter sua assinatura para o consentimento informado.

Os critérios de inclusão foram: adultos > 18 anos de idade em hemodiálise crônica por pelo menos 3 meses e dispostos a assinar o termo de consentimento livre e esclarecido. Os critérios de exclusão foram: indivíduos com marca-passo, válvulas cardíacas metálicas, amputações de membros, deficiência visual e analfabetismo. O tamanho da amostra foi calculado visando uma redução de 25% no ganho de peso interdialítico, considerando um nível alfa de 0,05 e um poder de 80%. A fórmula

utilizada foi $n={\left(\frac{Z\alpha }{2}+Z\beta \right)}^{2}*2*\sigma^{2}/\Delta^{2}$ onde *n*

é o tamanho da amostra, Z = 1,96 quando alfa é igual a 0,05, Zβ = 0,84 (valor normal para um poder de 80%), σ é o desvio padrão e delta é a diferença média entre os grupos.

### Espectroscopia de Bioimpedância

Para avaliar o estado volêmico, utilizamos um dispositivo de bioimpedância multifrequencial (*Body Composition Monitor* – BCM^®^ – Fresenius^®^ Medical Care, Deutschland GmbH, Alemanha).

### Tipo de Aprendizagem

Identificamos a forma preferida de aprendizagem de todos os pacientes incluídos no estudo por meio de uma ferramenta conhecida como VARK (*Visual* [Visual] - *Auditive* [Auditiva] – *Reading* [Leitura] – *Kinesthetic* [Cinestésica]). O questionário VARK é composto por 16 questões com 4 respostas possíveis. Cada resposta está associada a um tipo de preferência de aprendizagem e os respondentes podem escolher uma ou mais respostas para cada questão (ver arquivo suplementar para perguntas e respostas do questionário VARK). Agrupamos os participantes do estudo nas categorias V, A, R ou K com base no tipo de aprendizagem que obteve a pontuação mais elevada. Além disso, também classificamos os pacientes como tendo preferência bimodal caso selecionassem 2 dos 4 tipos de aprendizagem em mais de 25% das vezes e como tendo preferência multimodal se selecionassem 3 ou 4 tipos de aprendizagem (conforme mostrado na Figura 2).

**Figura 2:** Distribuição do escore VARK para cada tipo de aprendizagem.

### Medições de Pressão Arterial

A pressão arterial foi medida imediatamente antes da sessão de diálise, seguindo todas as recomendações de melhores práticas relacionadas ao posicionamento do paciente e ao tamanho do manguito, durante o período de estudo de 60 dias, sendo 30 dias na primeira fase e 30 na segunda fase. Não medimos a pressão arterial durante o período de *washout*. É importante ressaltar que o estudo ocorreu durante a pandemia de COVID-19, mas não houve ajustes ou alterações na duração e frequência da terapia durante esse período.

### Intervenção

Desenvolvemos quatro ferramentas para a educação individualizada do paciente com base no tipo principal de aprendizagem de cada indivíduo (conforme mostrado na Tabela 1). Cada sessão de treinamento durou de 20 a 30 minutos para cada paciente e foi realizada em duas sessões (durante a diálise), abordando temas como: conceitos relacionados à sobrecarga hídrica, importância de aderir às diretrizes nutricionais e orientações dietéticas sobre a ingestão de sódio e líquidos. No acompanhamento padrão, foi fornecida uma breve orientação nutricional verbal, sem o uso de materiais educativos, como um reforço em relação à restrição hídrica e de sódio (Figura 1).

**Tabela 1:** Material educacional

	Material developed
Visual	• Infográfico O tópico foi apresentado em formato de infográfico que enfatiza elementos visuais como imagens, formas, fluxogramas, realces de cores e visuais organizados com fontes e símbolos alternativos. O conteúdo foi apresentado em um tablet e também distribuído em formato de folheto com impressão colorida. • “FLASH CARDS”. Uma imagem é mostrada na frente do cartão e o paciente é solicitado a deduzir as informações no verso. Por exemplo, um alimento é exibido na frente, enquanto no verso, um sinal vermelho ou verde indica o teor de sódio (vermelho = alto e verde = baixo). Para líquidos, a frente mostra a foto de um líquido no recipiente e o verso fornece informações sobre a quantidade equivalente em copos de 200 ml.
Aural/auditivo	• Explicação de conceitos e orientações de forma oral. Durante a apresentação, perguntas sobre o tema são feitas ao paciente para incentivá-lo a falar e interagir. • Ao final, é realizado um teste oral com perguntas e respostas sobre as orientações fornecidas, para que o paciente possa repetir os conceitos em voz alta. • Uma mensagem de áudio sobre o assunto e as orientações (disponíveis em aplicativos de mensagens) é enviada para referência posterior.
Leitura/Escrita	• Orientações escritas são fornecidas em papel A4. Inicialmente apresentadas em um tablet e, em determinados momentos, o paciente é solicitado a ler alguns destaques em voz alta. • O material escrito é fornecido em papel A4 para ser lido pelo paciente. • No material escrito, são incluídas perguntas sobre o tema para que o paciente escreva as respostas, promovendo habilidades de leitura e escrita.
Cinestésico	• Apresentação de conceitos em PowerPoint, com uso de fotos e vídeos de situações de vida real para exemplificar alimentos/volume de líquidos, edema, etc. • Os rótulos dos alimentos são exibidos para avaliação e comparação do teor de sódio na tabela nutricional. São fornecidos exemplos de alimentos com baixo e alto teor de sódio. • Recipientes de diferentes tamanhos são utilizados para desenvolver o conceito de quantidade (xícara, caneca, tigela). Pede-se ao paciente que tente adivinhar quantos mL cada recipiente contém. • Na conclusão, com a participação do paciente, é calculado o número de copos de água necessários (hipoteticamente) para atingir o ganho de peso apresentado desde a última sessão de diálise.

### Estatísticas

As variáveis contínuas foram relatadas como média ± desvio padrão ou mediana e intervalo interquartil, dependendo da distribuição dos dados, e as variáveis categóricas como porcentagem e número absoluto. A comparação dos parâmetros clínicos entre os grupos (com base no tipo de intervenção) foi realizada por meio de regressão linear de efeitos mistos multinível, em que todas as medidas clínicas estavam no primeiro nível e o paciente no segundo nível. Utilizamos o STATA 17^®^ para todas as análises.

## Resultados

Nosso estudo incluiu 21 pacientes crônicos em hemodiálise, com idade média de 54,3 ± 14,4 anos, 24% do sexo masculino, 24% com diabetes e 81% com diagnóstico de hipertensão. Detalhes sobre todas as características demográficas e clínicas podem ser encontrados na Tabela 2.

**Tabela 2:** Características clínicas e demográficas da população do estudo

Variável	Resultado
Demográfica	
Idade (anos)	54,3 ± 14,4
Diabetes (sim)	23,8%
Hipertensão (sim)	80,9%
Renda	
1	19,0%
2	42,9%
3	28,6%
4	9,5%
Literacia	
1	28,6%
2	47,6%
3	23,8%
Sexo masculino	23,8%
Hemodiálise anterior (sim)	19,0%
Preferência VARK	
2	47,6%
3	14,3%
4	23,8%
5	14,3%
Clínica	
Tempo de diálise (meses)	20 (11–41)
PAS (mmHg)	166,6 ± 17,3
Ultrafiltração (litros)	2,7 ± 1,0
Laboratorial	
Potássio (mEq/L)	4,9 ± 0,8
Albumina (g/dl)	3,9 ± 0,4

A maioria dos pacientes apresentou uma combinação de respostas nos quatro tipos de aprendizagem, com muito poucos tendo mais de 50% de suas respostas dentro de um único tipo de aprendizagem (Figura 2). Apenas 7 pacientes obtiveram mais de 50% das respostas dentro de pelo menos um tipo de aprendizagem e 3 deles foram classificados como pacientes bimodais (1 visual/leitura, 1 auditivo/leitura e 1 auditivo/cinestésico). Se considerássemos somente a variável de maior valor para categorizar nossos sujeitos (independentemente do limite de 50%), teríamos: 10 pacientes como auditivos, 3 como leitura, 5 como cinestésicos e 3 com um tipo de aprendizagem bimodal.

A pressão arterial da população estudada mostrou-se elevada em comparação com os padrões atuais. Das 1.155 medições realizadas em todos os 21 pacientes, 91,9% estavam acima de 120 mmHg, 85,4% estavam acima de 130 mmHg e 74,5% estavam acima de 140 mmHg. A pressão arterial sistólica (PAS) correlacionou-se positivamente com o GPID (Figura 3), com um aumento de 4,5 mmHg para cada aumento de 1 litro no GPID. Em média, o GPID basal (3 primeiras medições) da população foi de 2,61 ± 1,08 litros (Figura S1) e não foi encontrada nenhuma alteração significativa tanto no GPID quanto na PAS ao longo do período de estudo (Figuras 4A e 4B). Além disso, nenhuma alteração relacionada à intervenção foi observada na PAD, no Kt/V da sessão e na hiper-hidratação (em litros) medida com bioimpedância multifrequencial (Tabela 3). Não houve alterações na prescrição de medicamentos anti-hipertensivos entre os grupos durante o período do estudo.

**Figura 3:** Correlação entre pressão arterial e GPID.

**Figura 4:** A. Ultrafiltração antes e após a intervenção por paciente; B. Ganho de peso intradialítico antes e após a intervenção por paciente.

Observações – A Figura A mostra os valores individuais de ultrafiltração durante o estudo, antes e após a intervenção. A figura B mostra os valores individuais do GPID durante o estudo, antes e após a intervenção.

**Tabela 3:** Parâmetros clínicos dos grupos com e sem educação individualizada

Parâmetros	Padrão	Individualizado	p
Pressão arterial sistólica pós-diálise (mmHg)	157,1 ± 17,2	159,5 ± 16,2	0,56
Pressão arterial sistólica pré-diálise (mmHg)	153,7 ± 16,3	152,0 ± 16,1	0,44
KtV	1,55 ± 0,56	1,61 ± 0,27	0,52
Ganho de peso interdialítico (kg)	2,48 ± 0,69	2,53 ± 0,62	0,55
Ultrafiltração por sessão (litros)	2,59 ± 0,71	2,70 ± 0,69	0,24
***Espectroscopia de bioimpedância***			
Hiper-hidratação (litros)	1,5 ± 2,0	1,41 ± 1,68	0,97

Também estratificamos nossa população para explorar nossos resultados de acordo com o tipo específico de aprendizagem (Figura 5). A intervenção foi considerada ineficaz, independentemente do tipo de aprendizagem.

**Figura 5:** Ganho de peso interdialítico antes e após a intervenção, estratificado por tipo de aprendizado.

Abreviações – A: auditiva; R: leitura; K: cinestésica. Nenhum paciente foi classificado como aprendiz visual.

## Discussão

Este estudo é o primeiro, até onde temos conhecimento, a testar a eficiência de uma orientação nutricional individualizada desenvolvida para diferentes tipos de aprendizagem na redução do GPID em pacientes crônicos em hemodiálise. Nossos resultados sugerem que essa ferramenta foi ineficaz na redução do GPID em curto prazo.

O GPID é um sinal clínico facilmente identificável na prática clínica e se correlaciona bem com a hipertensão arterial e a hipervolemia, que, por sua vez, são preditores conhecidos de eventos cardiovasculares e baixa qualidade de vida^4. Um estudo envolvendo uma grande coorte, com mais de 38.000 pacientes, constatou que a taxa de mortalidade entre os indivíduos no quartil mais alto do GPID foi mais de 2,5 vezes maior do que a do segundo quartil^5^. O grupo com a maior taxa de mortalidade apresentou um GPID superior a 4 kg, em comparação com 2,3 kg no grupo do segundo quartil. O GPID médio de nossa população situou-se entre o segundo e o terceiro quartil do estudo de Hecking et al.^5^ que apresentou um risco de óbito 17 a 20% maior. Essa característica de alto risco de nossa população nos levou a buscar uma estratégia para reduzir o GPID.^

O GPID é influenciado principalmente pela aderência do paciente às recomendações dietéticas da equipe médica e multidisciplinar em relação à ingestão de sódio e líquidos. Diante dessa premissa, vários autores enfatizam a importância de personalizar a abordagem para pacientes em diálise, a fim de minimizar o ganho de peso entre as sessões^6,7^. A ideia é que essa abordagem individualizada possa aumentar a adesão do paciente à terapia. No entanto, não há consenso sobre a melhor forma de personalizar essa recomendação.

Selecionamos o questionário VARK como uma ferramenta para transmitir conhecimento de forma personalizada. O instrumento foi aplicado pela primeira vez a um grupo de estudantes e possui uma confiabilidade estimada de 80%^8^. O sistema VARK categoriza os indivíduos com base em seu método preferido de receber novas informações - visual, auditivo, leitura/escrita, cinestésico ou multimodal - e mostrou-se útil quando aplicado às ciências médicas^9^. Em pacientes hipertensos com PA não controlada, foi obtido um melhor controle de longo prazo da PA após a intervenção individualizada utilizando o sistema VARK, com uma redução média de 9 mmHg na PA sistólica^10^. Em outro estudo com diabéticos, o grupo que utilizou a estratégia individualizada obteve maior sucesso no autocuidado relacionado à doença em comparação com a estratégia convencional^11^. No entanto, assim como o presente estudo, nem todas as pesquisas mostraram resultados positivos - Chan et al. não encontraram nenhuma melhora na aderência ao tratamento com o uso de um dispositivo visual para o manejo da asma em indivíduos com preferência de aprendizagem visual^12^.

As razões para os diferentes desfechos desses estudos com populações diversas não são imediatamente aparentes e exigem uma análise mais aprofundada. Por exemplo, nossos pacientes tinham uma condição crônica que leva a diversas limitações e, portanto, quebrar rotinas habituais é difícil com uma intervenção única. Além disso, outros fatores afetam a adesão ao tratamento, como sexo, nível educacional, tempo curto de diálise^10^, idade, baixa alfabetização em saúde e baixa qualidade de vida^13^. Um conhecimento limitado em saúde, de 22,7% (IC: 95% 20,6–24,8%), foi encontrado em uma revisão sistemática com 1.405 indivíduos, sendo independentemente associada a fatores socioeconômicos, como menor nível de escolaridade, renda e piores desfechos de saúde^14^. Como a literacia em saúde tem implicações importantes, as intervenções para o processo educacional requerem uma abordagem individualizada e contínua, bem como a consideração do estilo de aprendizagem específico do paciente. É provável que os resultados malsucedidos encontrados aqui se devam, em parte, à abordagem única do estudo. No entanto, é reconhecido na comunidade médica que nossa abordagem convencional (tamanho único para todos) precisa ser reavaliada^15^.

Uma meta-análise com 40 ensaios clínicos randomizados e um total de 2.278 pacientes mostrou que intervenções psicossociais e educacionais aumentaram as taxas de aderência de pacientes em hemodiálise e diálise peritoneal, e foram evidentes no GPID e na relação peso seco/GPID. As intervenções foram baseadas em diferentes contextos, como terapia cognitivo-comportamental, entrevista motivacional, relaxamento, apoio, educação, aconselhamento, consulta, fornecimento de recursos, supervisão e contato telefônico direto^16^. O estudo mostrou que a adesão ao tratamento está relacionada a múltiplos fatores que contribuem para que os pacientes adotem comportamentos, o que não foi possível observar em nosso estudo.

A abordagem para melhorar a adesão ao trata­mento em pacientes com doença renal crônica deve incluir conhecimento, comportamentos, ações e habilidades para capacitá-los a assumir a responsabilidade por seus próprios cuidados. Dessa forma, as informações fornecidas devem ser significativas, considerar seu estilo de aprendizagem e ser divididas em etapas que possam ser alcançadas por meio de objetivos pequenos e de curto prazo. Isso aumentará a confiança, de modo que o paciente se sinta capaz de dominar esse novo conhecimento e colocá-lo em prática^17^.

Nosso estudo apresentou algumas limitações importantes que devem ser consideradas, incluindo o fato de ter sido um estudo de centro único com um número pequeno de pacientes, a frequência da intervenção pode não ter sido suficiente para aumentar a conscientização e também o fato de que todos os pacientes já estavam em diálise, o que dificulta a alteração de seu comportamento, pois eles podem já estar seguindo uma determinada rotina. Além disso, somente uma pessoa treinada (uma nutricionista) realizou a intervenção, o que pode ser tanto positivo, já que não houve variação entre os observadores, quanto negativo, pois pode ter havido problemas com a pessoa que realizou a intervenção. Além disso, embora pacientes analfabetos tenham sido excluídos, não se sabe se o grau de alfabetização em saúde foi suficiente para compreender totalmente as orientações.

Em conclusão, o GPID continua sendo um problema sério na população em hemodiálise. Parece haver um consenso sobre a individualização do tratamento e da orientação nutricional, mas a melhor forma de individualizar o tratamento ainda é desconhecida. A individualização do tratamento com base no estilo de aprendizagem do paciente não melhorou o GPID em nossa população.

## Material Suplementar

O seguinte material online está disponível para este artigo:

Questionário VARK.

Figura S1 – GPID no início do estudo.
